# Systemic effects of intratympanic dexamethasone therapy

**DOI:** 10.1530/EC-14-0076

**Published:** 2014-08-14

**Authors:** Eva Novoa, Marcel Gärtner, Christoph Henzen

**Affiliations:** 1 Department of Otorhinolaryngology Head and Neck Surgery; 2 Clinic of Endocrinology Diabetology and Clinical Nutrition, Department of Internal Medicine, Kantonsspital Luzern, CH-6000 Luzern 16 Switzerland

**Keywords:** intratympanic, dexamethasone, acute hearing loss, Ménière's disease, cortisol, osteocalcin

## Abstract

**Objective:**

The study aimed to assess the possible systemic effects of intratympanic dexamethasone (IT-Dex) on the hypothalamic–pituitary–adrenal (HPA) axis, inflammation, and bone metabolism.

**Design:**

A prospective cohort study including 30 adult patients of a tertiary referral ENT clinic treated with 9.6 mg IT-Dex over a period of 10 days was carried out.

**Methods:**

Effects on plasma and salivary cortisol concentrations (basal and after low-dose (1 μg) ACTH stimulation), peripheral white blood cell count, and biomarkers for bone turnover were measured before (day 0) and after IT-Dex (day 16). Additional measurements for bone turnover were performed 5 months after therapy. Clinical information and medication with possible dexamethasone interaction were recorded.

**Results:**

IT-Dex was well tolerated, and no effect was detected on the HPA axis (stimulated plasma and salivary cortisol concentration on day 0: 758±184 and 44.5±22.0 nmol/l; day 16: 718±154 and 39.8±12.4 nmol/l; *P*=0.58 and 0.24 respectively). Concentrations of osteocalcin (OC) and bone-specific alkaline phosphatase (BSAP) did not differ after dexamethasone (OC on days 0 and 16 respectively: 24.1±10.5 and 23.6±8.8 μg/l; BSAP on day 0, 16, and after 5 months respectively: 11.5±4.2, 10.3±3.4, and 12.6±5.06 μg/l); similarly, there was no difference in the peripheral white blood cell count (5.7×10^12^/l and 6.1×10^12^/l on days 0 and 16 respectively).

**Conclusions:**

IT-Dex therapy did not interfere with endogenous cortisol secretion or bone metabolism.

## Introduction

Intratympanic glucocorticoids are effective in the treatment of sudden sensorineural hearing loss (SSNHL) (1, 2, 3), in the stabilization of Ménière's disease (MD) (4), and in autoimmune ear disorders (5). By the intratympanic route of administration, the varied systemic side effects of oral or intravenous glucocorticoids should be minimized, in particular the suppression of the hypothalamic–pituitary–adrenal (HPA) axis or the inhibition of osteoblast function. Even after short-term systemic prednisolone treatment for at least 5 days, a partial inhibition of the adrenal function results in ∼40% of the patients (6), exposing them to the risk of an Addisonian crisis in high-stress situations such as critical illness or surgery. Furthermore, osteoporosis is a well-known side effect of systemic glucocorticoids, and short-term cumulative doses of 80–160 mg of methylprednisolone may also cause osteonecrosis (7), a commonly overlooked complication.

In the treatment of SSNHL, a broad range of therapeutic modalities are being explored with different doses and applications of glucocorticoids that usually by far exceed the physiological daily cortisol production (8), and therefore carry the risk of systemic side effects. A large multicentre study comparing the efficacy of intratympanic vs systemic steroid therapy demonstrated the same level of hearing improvement between the two groups (9). However, for the time being, there is no consensus regarding the dose and frequency of the glucocorticoid treatment of this disorder. In animal studies, intratympanic dexamethasone (IT-Dex) infusions resulted in higher perilymph concentrations than intravenous dexamethasone application without any systemic absorption (10). By contrast, recently published studies have detected small concentrations of methylprednisolone in human blood samples after intratympanic application (11) generating some controversy about systemic effects of intratympanic glucocorticoids.

We therefore aimed to assess the possible systemic effects of IT-Dex injections on the HPA axis and bone metabolism, particularly on osteoblast function, as highly sensitive cells to exogenous glucocorticoids, in a prospective cohort study of patients receiving IT-Dex treatment.

## Materials and methods

### Participants

In our tertiary care hospital, a prospective study was carried out including adult patients with SSNHL or severe MD. All patients received IT-Dex treatment. Exclusion criteria were the following: inability to provide written consent, pregnancy or lactation, patients younger than 18 years, a history of systemic glucocorticoid treatment during the last 6 months, and a history of pituitary or adrenal disorders and liver disease.

Local ethics committee approved the study protocol and an informed consent was obtained from each participant.

### IT-Dex therapy

IT-Dex was performed as described previously (3): a total of four intratympanic applications were performed within 10–12 days with 2–3 days interval between each application. Each dose consisted of a volume of 0.5 ml containing a mixture of 0.3 ml dexamethasone dihydrogenphosphate (8 mg/ml) and 0.2 ml hyaluronic acid (0.2%). The solution was prepared by the pharmacy of our hospital and kept at 4 °C in 1 ml syringes for single use. The procedure was performed under local anesthesia. While the patient was in supine position, the mixture was slowly injected into the middle ear with a 23-gauge needle placed anterosuperior under microscopic view. After the injection, the patient was asked to turn his head 45° to the opposite side and to remain in this position for 30 min, avoiding to swallow and to speak.

### Plasma and salivary cortisol measurements

**Figure 1:**
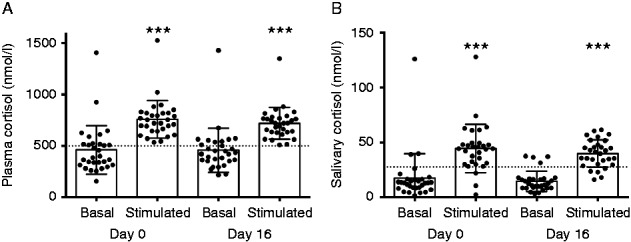
Low-dose (1 μg) ACTH-stimulation test in 30 patients with intratympanic dexamethasone treatment: plasma (A) and salivary (B) cortisol concentrations on days 0 (before IT-Dex) and 16 (after IT-Dex therapy). Data are expressed as median (bars)± standard deviation (whiskers), and outliers. Statistical significance between stimulated and basal cortisol levels is denoted by asterisks, *P*<0.001.

**Figure 2:**
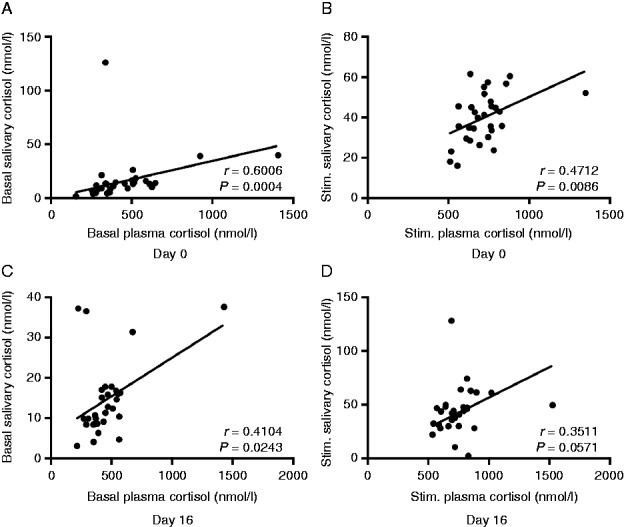
Correlation between plasma and salivary cortisol concentrations on days 0 (A, B) and 16 (C, D), respectively, in 30 patients receiving intratympanic dexamethasone treatment.

**Figure 3:**
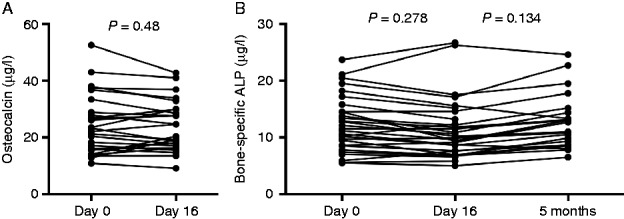
Effect of intratympanic dexamethasone treatment on markers of bone metabolism in 30 patients on days 0 and 16, and in 20 patients at 5 months: osteocalcin (A) and bone-specific alkaline phosphatase (B).

Measurements of basal plasma and salivary cortisol concentrations were performed immediately before IT-DEX (day 0), and 6 days after the last IT-Dex application, i.e. day 16 or 18 (referred to as day 16 in Figs 1, 2 and 3). This period of time was chosen in order to identify possible adrenal insufficiency after the dexamethasone therapy.

Low-dose (1 μg) ACTH (1-24)-corticotrophin was injected in order to generate a physiological adrenocortical stimulation (12), providing higher sensitivity and specificity in the assessment of the function of the HPA axis than the conventional standard 250 μg Synacthen test (13). One vial of 250 μg tetracosactrin (Synacthen, Novartis Pharma, Berne, Switzerland) was diluted in sterile saline solution to a concentration of 1 μg/ml, filtered in plastic syringes, and stored at 4 °C. The tests were carried out according to the local protocol.

Plasma and salivary cortisol levels were measured using a commercially available chemiluminescent immunoassay Cobas 6000 analyzer (Roche) according to the manufacturer's procedure. The interassay coefficient of variation was 4.5%. Thirty minutes after the injection of 1 μg ACTH, blood and saliva samples were taken to measure stimulated plasma and salivary cortisol concentrations respectively. The low-dose (1 μg) ACTH stimulation tests were carried out between 0800 and 0900. Samples were collected and analyzed immediately. Salivary samples were collected in Salivette (14). Normal responses to 1 μg ACTH were defined as stimulated plasma cortisol concentrations >500 nmol/l and/or a cortisol increase of at least 250 nmol/l, and as stimulated salivary cortisol concentrations >27.5 nmol/l.

### Markers of bone metabolism and inflammation

The following additional blood samples of each patient were obtained on days 0 and 16 respectively: white blood cell count, osteocalcin (OC), and bone-specific alkaline phosphatase (BSAP). In order to evaluate any persistence of changes in BSAP over time, another blood test was carried out during outpatient clinical control 5 months after completing IT-Dex. Temperature-labile blood samples for OC were immediately sent for centrifugation and frozen until analyses were performed. The normal range for OC was considered to be between 8.8 and 29.7 μg/l for men and 5.2 and 34.5 μg/l for women. The normal range for BSAP in males was 6–30 μg/l, in premenopausal women 3–19 μg/l, and in postmenopausal women 6–26 μg/l. The plasma concentrations of OC and BSAP were measured by electrochemiluminescence immunoassays using a LIAISON Analyzer (DiaSorin, Inc., Stillwater, OK, USA) for BSAP and by chemiluminescence immunoassay (Immunodiagnostic Systems Ltd, Boldon, England) for OC. The interassay coefficient of variation was 8.7% for BSAP and 6.05% for OC.

### Follow-up

Patients were interviewed on the day of the first IT-Dex application (day 0) and 6 days (day 16) after completion of the therapy. A detailed questionnaire relating to possible systemic side effects was completed by each participant after IT-Dex including mood changes, infections, particularly middle ear infections, trauma or surgery, and possible drug interactions with dexamethasone (i.e. antidiabetic, anticonvulsants, oral anticoagulants, antihypertensives, antidepressants, NSAID, and statins), as well as blood glucose control and blood pressure.

### Statistical analysis

Statistical analyses were performed using GraphPad Prism version 6.00 for Mac OS X, GraphPad Software, La Jolla, CA, USA (www.graphpad.com). Data are expressed as means and s.d. unless stated otherwise. The paired *T*-test was used to assess normally distributed data (checked by the Kolmogorov–Smirnov test). The non-parametric Mann–Whitney test was applied otherwise. Relations between variables were analyzed by linear regression and Spearman's correlation analysis. A *P* value of <.05 was considered statistically significant in the above tests.

## Results

### Participants

The study included 30 (ten females and 20 males) consecutive patients admitted to our ENT clinic with a mean age of 60.1±9.8 years. Baseline characteristics are given in Table 1: 25 (83%) patients presented with relevant comorbid conditions (13 patients (43%) had one comorbidity, six patients (20%) had two comorbidities, and six patients (20%) had three or more comorbidities), and there were nine patients (30%) who had medical treatment interacting with dexamethasone, i.e. attenuating the efficacy by inducing dexamethasone metabolism in seven patients (23%) (e.g. anticonvulsants or oral anticoagulants) and enhancing the therapeutic effects in two patients (6%) (e.g. NSAID). Before and after IT-Dex, the well-being index, blood pressure, and fasting plasma glucose were similar with no statistically significant difference, and there were no infections observed.

### Plasma and salivary cortisol measurements

Basal and stimulated plasma cortisol concentrations were 461±236 and 758±184 nmol/l on day 0 (*P*<0.001), i.e. before IT-Dex, and 458±214 and 718±154 nmol/l on day 16 (*P*<0.001), i.e. after IT-Dex (*P*=0.91 and 0.58 for basal and stimulated plasma cortisol concentrations respectively). Similarly, there was no significant difference in basal and stimulated salivary cortisol levels before and after IT-Dex: 17.4±22.3 and 44.5±22.0 nmol/l on day 0, and 14.6±9.3 and 39.8±12.4 nmol/l on day 16 respectively (*P*=0.85 and 0.24 for basal and stimulated salivary cortisol concentrations respectively). Fig. 1 summarizes the results of the low-dose (1 μg) ACTH stimulation test, demonstrating that all patients who met the laboratory criteria for normal adrenal function before IT-Dex also had normal stimulation tests after IT-Dex: five patients had near-normal or low stimulated salivary cortisol concentrations before and after IT-Dex, though, their basal and stimulated plasma cortisol concentrations were in the normal range suggesting preanalytical problems in collecting adequate amounts of saliva resulting in falsely low salivary cortisol levels.

Plasma and salivary cortisol levels yielded excellent correlation, even without correction for low basal salivary cortisol concentrations that presumably were caused by sampling errors: basal and stimulated plasma and salivary cortisol levels were positively correlated on days 0 and 16 (Fig. 2).

### Markers of bone metabolism and inflammation

OC concentrations on day 0 were 24.1±10.5 μg/l and 23.6±8.8 μg/l on day 16, with no statistically significant difference before and after IT-Dex (*P*=0.48). Similarly, levels of BSAP were 11.51±4.16 and 10.29±3.45 μg/l on days 0 and 16 respectively (*P*=0.278). Five months later, levels of BSAP (*n*=20) were 12.59±5.059 μg/l. No significant differences in BSAP were observed between day 0 and month 5 (*P*=0.558) and between day 16 and month 5 (*P*=0.134). Well-known age- and gender-related differences in OC and BSAP concentrations were observed in our cohort. The bone biomarker data are summarized in Fig. 3.

The WBC remained within normal values on days 0 and 16, i.e. 5.7×10^12^/l and 6.1×10^12^/l respectively, and there were no changes in the number of eosinophils before and after IT-Dex (0.099×10^12^/l, and 0.178×10^12^/l respectively; *P*=0.20).

## Discussion

This study demonstrated that IT-Dex therapy in patients with SSNHL or severe MD does not adversely affect adrenal function or markers of bone metabolism. Intratympanic glucocorticoid application proved to be an effective treatment of SSNHL mainly because of higher endo- and perilymphatic glucocorticoid concentration compared with systemic glucocorticoid administration (2, 3, 6), with the presumed advantage of fewer side effects. However, there is some controversy about possible systemic side effects of the intratympanic glucocorticoid application (15). To clarify this issue, we assessed the HPA axis and markers of osteoblast function as two highly glucocorticoid-sensitive endogenous systems revealing even minor interferences with exogenous glucocorticoid application. In contrast to previous reports describing an inhibition of the HPA axis after a prolonged near-continuous transtympanic steroid perfusion of the inner ear (via a permanent tube to deliver treatment fluids to the middle ear such as the Silverstein MicroWick) (15), our results demonstrated no difference in the function of the HPA axis and of marker of bone metabolism before and after four single applications of IT-Dex, and there was no difference in WBC or eosinophil count. Thus, systemic side effects of the widely implemented method of administration of glucocorticoids based on single dexamethasone applications with well-defined treatment intervals can reliably be excluded. We hypothesize that the possible clearance and systemic resorption of the dexamethasone through the middle ear mucosa and its elimination through the Eustachian tube (16, 17), after the equilibrium between both sides of the round window membrane is accomplished, do not result in any clinically meaningful dexamethasone concentration in the circulation. Even though the total amount of dexamethasone reaching the inner ear after IT-Dex is high (9.6 mg over a time period of 10 days), this treatment modality of SSNHL seems to be safe considering systemic side effects. This favorably contrasts to other therapeutic injection sites where systemic glucocorticoid effects could be demonstrated, e.g. epidural triamcinolone injections causing suppression of the HPA axis in over 70% of the patients with lumbar disc herniation (18). Patients with SSNHL often suffer from concomitant diseases such as hypertension, diabetes, cardiovascular disease, or depression, which most probably would deteriorate under systemic glucocorticoid impact. Further, in a high proportion of these patients with pre-existing medication, drug interactions with dexamethasone are a potential issue that can be minimized by the localized effect of IT-Dex injections.

In conclusion, our study demonstrates that IT-Dex therapy does not cause systemic side effects, i.e. our findings confirm the normal function of the HPA axis and the osteoblasts after intratympanic application.

## Figures and Tables

**Table 1:** Baseline characteristics of patients receiving intratympanic dexamethasone therapy (*n*=30). Age (years): 60.1±9.8; sex (M/F, patients, *n* (%)): 20 (66%)/10 (33%)

**Comorbidity**	**Patients**, ***n*** (%)	**Medication**	**Patients**, ***n*** (%)
Coronary heart disease	5 (17%)	Antihypertensives	
		ACE Inhibitors	8 (27%)
	Betablockers	5 (17%)
Depression	6 (20%)	Anticonvulsants	3 (10%)
Diabetes mellitus	3 (10%)	Antidepressants	2 (7%)
Dyslipidemia	2 (7%)	NSAID	2 (7%)
Epilepsy	3 (10%)	Oral anticoagulants	4 (13%)
Hypertension	8 (27%)	Statins	8 (27%)
Osteoporosis	4 (13%)		

## References

[1] Plontke SK, Biegner T, Kammerer B, Delabar U, Salt AN (2008). Dexamethasone concentration gradients along scala tympani after application to the round window membrane. Otology & Neurotology.

[2] Rauch SD, Halpin CF, Antonelli PJ, Babu S, Carey JP, Gantz BJ, Goebel JA, Hammerschlag PE, Harris JP, Isaacson B (2011). Oral vs intratympanic corticosteroid therapy for idiopathic sudden sensorineural hearing loss: a randomized trial. JAMA.

[3] Burkart C, Linder T, Gartner M (2013). Intratympanic steroid administration: use in the treatment of profound idiopathic sudden sensorineural hearing loss. HNO.

[4] Phillips JS, Westerberg B (2011). Intratympanic steroids for Meniere's disease or syndrome. Cochrane Database of Systematic Reviews.

[5] Hu A, Parnes LS (2009). Intratympanic steroids for inner ear disorders: a review. Audiology & neuro-otology.

[6] Henzen C, Suter A, Lerch E, Urbinelli R, Schorno XH, Briner VA (2000). Suppression and recovery of adrenal response after short-term, high-dose glucocorticoid treatment. Lancet.

[7] Weinstein RS (2012). Glucocorticoid-induced osteonecrosis. Endocrine.

[8] Seggas I, Koltsidopoulos P, Bibas A, Tzonou A, Sismanis A (2011). Intratympanic steroid therapy for sudden hearing loss: a review of the literature. Otology & Neurotology.

[9] Bae SC, Noh HI, Jun BC, Jeon EJ, Seo JH, Park SY, Kim JK, Lee DH, Oh JH, Park SN (2013). Efficacy of intratympanic steroid therapy for idiopathic sudden sensorineural hearing loss: comparison with systemic steroid therapy and combined therapy. Acta oto-Laryngologica.

[10] Chandrasekhar SS, Rubinstein RY, Kwartler JA, Gatz M, Connelly PE, Huang E, Baredes S (2000). Dexamethasone pharmacokinetics in the inner ear: comparison of route of administration and use of facilitating agents. Otolaryngology--Head and Neck Surgery.

[11] Bird PA, Begg EJ, Zhang M, Keast AT, Murray DP, Balkany TJ (2007). Intratympanic versus intravenous delivery of methylprednisolone to cochlear perilymph. Otology & Neurotology.

[12] Rasmuson S, Olsson T, Hagg E (1996). A low dose ACTH test to assess the function of the hypothalamic–pituitary–adrenal axis. Clinical Endocrinology.

[13] Giordano R, Picu A, Bonelli L, Balbo M, Berardelli R, Marinazzo E, Corneli G, Ghigo E, Arvat E (2008). Hypothalamus–pituitary–adrenal axis evaluation in patients with hypothalamo-pituitary disorders: comparison of different provocative tests. Clinical Endocrinology.

[14] Poll EM, Kreitschmann-Andermahr I, Langejuergen Y, Stanzel S, Gilsbach JM, Gressner A, Yagmur E (2007). Saliva collection method affects predictability of serum cortisol. Clinica Chimica Acta.

[15] Robey AB, Morrow T, Moore GF (2010). Systemic side effects of transtympanic steroids. Laryngoscope.

[16] Salt AN, Hartsock J, Plontke S, Lebel C, Piu F (2011). Distribution of dexamethasone and preservation of inner ear function following intratympanic delivery of a gel-based formulation. Audiology & neuro-otology.

[17] Salt AN, Plontke SK (2009). Principles of local drug delivery to the inner ear. Audiology & neuro-otology.

[18] Casutt M, Tovias H, Cristoph H, Christoph K, Helmut G, Guido S (2010). Suppression of the hypothalamic–pituitary–adrenal axis after epidural glucocorticoid injection: identification of inherent at-risk patients. Journal of Pain Management.

